# Duration of SARS-CoV-2 sero-positivity in a large longitudinal sero-surveillance cohort: the COVID-19 Community Research Partnership

**DOI:** 10.1186/s12879-021-06517-6

**Published:** 2021-08-30

**Authors:** The COVID-19 Community Research Partnership Study Group, David M. Herrington, John W. Sanders, Thomas F. Wierzba, Martha Alexander-Miller, Mark Espeland, Alain G. Bertoni, Allison Mathews, Austin L. Seals, Iqra Munawar, Michael S. Runyon, Lewis H. McCurdy, Michael A. Gibbs, Karen Kotloff, DeAnna Friedman-Klabanoff, William Weintraub, Adolfo Correa, Diane Uschner, Sharon Edelstein, Michele Santacatterina

**Affiliations:** Section on Cardiovascular Medicine, Department of Medicine, Wake Forest University School of Medicine, Medical Center Blvd., Winston Salem, NC 27157 USA

**Keywords:** COVID-19, Sero-surveillance, Humoral response

## Abstract

**Background:**

Estimating population prevalence and incidence of prior SARS-CoV-2 infection is essential to formulate public health recommendations concerning the COVID-19 pandemic. However, interpreting estimates based on sero-surveillance requires an understanding of the duration of elevated antibodies following SARS-CoV-2 infection, especially in the large number of people with pauci-symptomatic or asymptomatic disease.

**Methods:**

We examined > 30,000 serology assays for SARS-CoV-2 specific IgG and IgM assays acquired longitudinally in 11,468 adults between April and November 2020 in the COVID-19 Community Research Partnership.

**Results:**

Among participants with serologic evidence for infection but few or no symptoms or clinical disease, roughly 50% sero-reverted in 30 days of their initial positive test. Sero-reversion occurred more quickly for IgM than IgG and for antibodies targeting nucleocapsid protein compared with spike proteins, but was not associated with age, sex, race/ethnicity, or healthcare worker status.

**Conclusions:**

The short duration of antibody response suggests that the true population prevalence of prior SARS-CoV-2 infection may be significantly higher than presumed based on earlier sero-surveillance studies. The impact of the large number of minimally symptomatic COVID-19 cases with only a brief antibody response on population immunity remains to be determined.

**Supplementary Information:**

The online version contains supplementary material available at 10.1186/s12879-021-06517-6.

## Background

Determining the proportion of the population previously infected with SARS-CoV-2 and how this rate has changed over time is essential to understand the pandemic and recommendations for clinical preparedness, physical distancing, targeting of vaccines, and resumption of economic activities. Unfortunately, tests for viral antigens or RNA in symptomatic or high-risk individuals are inadequate for this purpose because of the transient nature of viral shedding.

Sero-surveillance, especially when deployed in large, population-based samples is thought to provide more accurate estimates of the prevalence of prior SARS-CoV-2 infection. Indeed, several sero-surveillance studies have highlighted the fact that a significant proportion of previously infected people are pauci- or completely asymptomatic and therefore likely missed by clinically motivated testing [1, 2]. These data illustrate the importance of using testing strategies that include minimally and asymptomatic cases when estimating community transmission.

However, sero-surveillance for SARS-CoV-2 infection has important limitations. In addition to the well described issues related to the sensitivity and specificity of different serologic assays [3, 4] questions still remain about the expected duration of elevated antibodies following SARS-CoV-2 infection. Understanding the dynamics of the humoral response is important as it has a direct impact on completeness of ascertainment when using sero-surveillance to determine population prevalence. The durability of the humoral response may also provide clues concerning the degree of immune activation following primary infections and the likelihood of subsequent long-term immunity in individuals and in the population. Preliminary evidence from small clinical studies suggests that minimally symptomatic infections often have an attenuated antibody response [3, 5–10]; however, more data are needed from large population samples with more detailed information on symptoms to complement the data from these intensive laboratory-based investigations.

Accordingly, we examined more than 30,000 longitudinally acquired serology test results from more than 11,461 adults enrolled in the COVID-19 Community Research Partnership—a population-based COVID-19 syndromic and sero-surveillance study based in two large healthcare systems in central North Carolina. The overwhelming majority of participants had few or no symptoms of COVID-19 even though more than 10% had serologic evidence of infection. Thus, this study provides a unique opportunity to examine the durability of antibody responses in a population-based survey including the large and critically important portion of the population with asymptomatic or pauci-symptomatic infection.

## Methods

Beginning on April 16th, 2020 potential participants 18 years and older identified in the Wake Forest Baptist Health (WFBH) and the Atrium Health (AH) systems were invited to participate through email, internal communications, websites, and social and general media. All participants provided informed consent for participation in the study and all methods were carried out in accordance with the relevant guidelines and recommendations concerning the conduct of clinical research. The protocol and informed consent was reviewed and approved by the Wake Forest School of Medicine Institutional Review Board.

Participants were asked to record daily symptoms (e.g., fever, cough, shortness of breath, etc.) related to COVID-19 [11] using a web-based Patient Monitoring System application (Oracle Corporation, Redwood Shores, California). A subset of participants (serology cohort) was also selected for longitudinal sero-surveillance based on their age, race, and gender in an effort to match the distribution of these demographics in their county of residence [12], with oversampling of certain high-risk groups (health care workers and minorities).

Most participants selected for sero-surveillance were mailed kits for in-home testing of finger-prick capillary blood. The kits provide written, video and audio instructions on how to clean and prick their finger with the provided lancet, collect the required 20 uL of blood with the collection tube, add the blood and diluent on the test cassette and take a photo of the result after 13 min of development time. Any evidence of a visible purple line in the region of the IgG and IgM capture region was considered positive, if also accompanied by a positive control line. Initially participants received a Syntron/Tianjin New Bay Bioresearch lateral flow assay (LFA) to test for IgM and IgG antibodies to the SARS-CoV-2 nucleocapsid antigens (n = 13,752 assays). In-home LFA results were recorded and interpreted via a smartphone application with central review (Scanwell Health, Inc. © 2020). A subset of participants received instead two 20 µL volumetric absorptive microsamplers (Mitra®, Neoteryx) for blood collection that were returned by mail analyzed centrally. To elute antibody, tips were placed in 100 µl of elution buffer (PBS + 1% BSA + 0.5% Tween 20), shaken on an orbital shaker (150 rpm) for 20–24 h at room temperature and then spun for 5 min @ 4000 rpm. The 20 ul of eluent was then loaded onto the Syntron LFA cassette and interpreted identically to the capillary blood specimens (n = 4313 assays). In July, 2020 the Syntron assay became unavailable after which participants received the Teco Diagnostics LFA to test for IgM and IgG antibodies to the SARS-CoV-2 spike and nucleocapsid antigens (n = 16,868 assays). Both assays were validated at the Frederick National Laboratory for Cancer Research (FNLCR) by the National Cancer Institute (NCI) using a panel of antibody-positive samples from patients with Polymerase Chain Reaction (PCR)-confirmed SARS-CoV-2 infection or pre-pandemic controls (Panel 2) [13]:Syntron (Tianjin New Bay): (antibody: sensitivity/specificity); IgM: 93.3%/98.8%; IgG:93.3%/98.8%; IgM or IgG:100%/97.5%) [14] Teco Diagnostics: (antibody: sensitivity/specificity); IgM:86.2%/99.0%; IgG: 84.5%/99.0%; IgM or IgG:93.1%/97.9% [13, 15].

The number and cadence of tests performed by each participant was influenced by the rolling enrollment into the cohort over time (earlier enrollees had more time for serial testing), as well as several factors related to the pandemic including interruptions in supply chains and test kit availability, shipping delays to and from the participants, and variability in the rate participants completed in-home tests or returned specimens for in-lab testing. Thus, estimates of sero-reversion in this report are derived from samples of the entire seroconversion cohort over a range of times following an initial positive test rather than assessment of the entire cohort at precisely timed intervals. The number and cadence of testing was similar among those with at least one positive test during follow-up and those that remained negative (Additional file 1: Figure S1).

Conventional parametric measures of central tendency and variance were used unless the distribution suggested that other approaches (e.g. Poisson confidence intervals) were more suitable. Logistic regression was used to estimate the relative odds of seroconversion as a function of symptom prevalence (JMP Ver. 15.0, SAS Institute). Multivariable Weibull [16] and semi-parametric Cox proportional hazard [17] models for interval-censored data were used to estimate the survival curve of time to sero-reversion controlling for age, self-reported, race/ethnicity, healthcare worker status, and enrolling healthcare system. The Wald test based on bootstrap standard errors was used for significance testing of the parameter estimates. (R package icenReg, v 3.63 [18]).

Role of the funding source: This work was supported by a grant from the State of North Carolina funded by the CARES Act, of the U.S. Department of Health and Human Services (HHS). The sponsor had no role in the developing the study design; in the collection, analysis, and interpretation of data; in the writing of the report; or in the decision to submit the paper for publication.

## Results

**Fig. 1:**
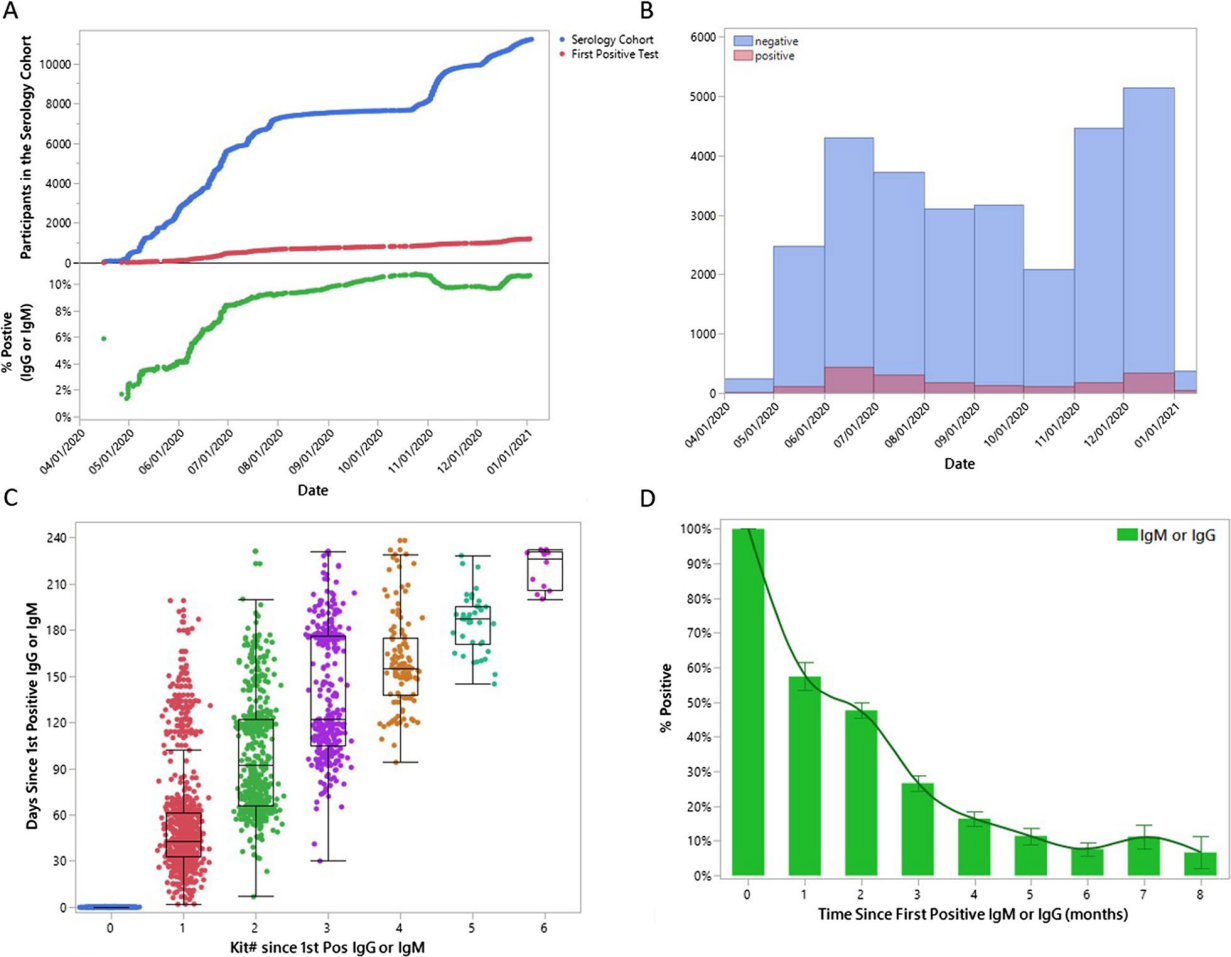
Distribution of enrollment of participants and serology tests as a function of time. **A** Number of participants enrolled (blue line—upper pane), and number (red line—upper pane) and percent (green line—lower pane) of participants who seroconverted from April 4th 2020 to Jan. 9th 2021. By Jan 2021 roughly 10% of the serology cohort had seroconverted. **B** Number of positive and negative tests from April 4th 2020 to Jan. 9th 2021. The monthly test positive rate roughly reflects the rate of community transmission in the study cohort as a function of time. **C** Distribution of longitudinal tests following an initial positive result. Each dot represents an individual test plotted on the y-axis based on time since the first positive test (indicated as kit# 0 on the x-axis). As of Jan 4, 2021, 11 participants had completed 6 tests following an initial positive test. **D** Percent of positive tests as a function of time following initial seroconversion

Between April 16th, 2020 and Jan. 4th, 2021 11,468 participants aged 18–94 years. completed a total of 30,620 serologic tests for IgM or IgG antibodies to SARS-CoV-2 antigens (tests/participant: range: 1–8; mean ± 95%CI_(Poisson)_ 2.67 ± 2.64–2.70; Table 1, Fig. 1).

**Table 1:** Participants in the sero-survey

	Serology cohort			Seropositive sub-cohort		
	(n = 11,468)			(n = 1172)		
Total number of tests	30,620			3856*		
	n	%	Tests/person (mean)	n	%	Tests/person (mean)
*Age (years)*						
1. < 30	1003	8.7%	2.4	99	8.5%	3.0
2. 30–39	2357	20.6%	2.8	258	22.0%	3.3
3. 40–49	2420	21.1%	2.8	244	20.8%	3.4
4. 50–59	2486	21.7%	2.7	258	22.0%	3.4
5. 60–69	2079	18.1%	2.6	210	17.9%	3.3
6. > = 70	1123	9.8%	2.5	103	8.8%	3.0
*Sex*						
F	7085	61.8%	2.7	719	61.4%	3.3
M	4383	38.2%	2.7	453	38.7%	3.3
*Race/ethnicity*						
Black or African American	622	5.4%	2.1	71	6.1%	2.2
Hispanic or Latino	351	3.1%	2.2	42	3.6%	2.4
Other	554	4.8%	2.4	55	4.7%	2.9
White (not Hispanic/Latino)	9941	86.7%	2.7	1004	85.7%	3.4
*Healthcare worker*						
N	6949	60.6%	2.4	629	53.7%	3.1
Y	4519	39.4%	3.1	543	46.3%	3.5
*Healthcare system*						
Atrium Health	2589	22.6%	2.5	298	25.4%	2.9
Wake Forest Baptist Health	8879	77.4%	2.7	874	74.6%	3.4

*Including first positive and all subsequent tests

During the period of observation 1172 people had at least one positive test for either IgG or IgM (crude sero-prevalence = 10.2%). Active daily symptom monitoring beginning at enrollment confirmed that COVID-19 symptoms were uncommon in this seropositive cohort. A COVID-like illness (defined as fever plus cough or shortness of breath for two out of three consecutive days) in the month prior to serology testing was associated with a positive result (OR = 11.4, p < 0.0001); but was reported in only 4% of seropositive participants. Similarly, two of three consecutive days of fever, sore throat, cough, shortness of breath, chest pain, muscle pain, nausea, diarrhea, headache, or anosmia were individually associated with subsequent seroconversion when present (all p ≤ 0.0004), but were infrequently reported (symptom prevalence range: 1–17%). Seventy-two percent (72%) of participants did not report a single day of symptoms prior to their first positive test.

A small number of participants (n = 56) reported a clinical diagnosis of COVID-19 prior to enrollment which was confirmed with their initial serology test. Another 13 participants developed symptomatic COVID-19 requiring hospitalization during follow-up. Collectively, these cases of clinically significant COVID-19 represent 6% of the seropositive cohort.

Of the 1172 people with at least one positive test for either IgM or IgG, 770 participants had 1–6 additional tests over the following eight months (mean interval between tests = 47.8 days, Fig. 1C). Among the 148 participants who completed their next test within 30 days only 85/148 (57%) remained positive for either IgG or IgM (Table 2). The percent of positive tests from the seropositive cohort continued to decline to < 10% over the next five months. A similar early decline in sero-positivity was observed when examining results for the IgM or the IgG assays individually.

**Table 2:** Test results as a function of time following an initial positive result

	Baseline*	Month 1		Month 2		Month 3		Month 4		Month 5		Month 6		> 6 Months	
	*n*	*n*	%	*n*	%	*n*	%	*n*	%	*n*	%	*n*	%	*n*	%
*Seropositive cohort*															
IgM															
Negative	0	59	48.8%	257	61.8%	255	78.7%	227	88.0%	155	93.4%	168	92.3%	99	93.4%
Positive	973	62	51.2%	159	38.2%	69	21.3%	31	12.0%	11	6.6%	14	7.7%	7	6.6%
IgG															
Negative	0	23	41.1%	73	38.6%	57	57.6%	79	71.2%	34	75.6%	38	95.0%	17	70.8%
Positive	532	33	58.9%	116	61.4%	42	42.4%	32	28.8%	11	24.4%	2	5.0%	7	29.2%
IgG or IgM															
Negative	0	63	42.6%	255	52.4%	264	73.3%	254	83.6%	163	88.6%	181	92.3%	107	89.9%
Positive	1172	85	57.4%	232	47.6%	96	26.7%	50	16.4%	21	11.4%	15	7.7%	12	10.1%
IgG and IgM															
Negative	0	19	65.5%	74	64.4%	44	71.0%	52	81.3%	23	85.2%	25	96.2%	8	72.7%
Positive	330	10	34.5%	41	35.7%	18	29.0%	12	18.8%	4	14.8%	1	3.9%	3	27.3%
*Subset with initial positive IgG and IgM*															
IgG and IgM															
Negative	0	19	65.5%	74	64.4%	44	71.0%	52	81.3%	23	85.2%	25	96.2%	8	72.7%
Positive	330	10	34.5%	41	35.7%	18	29.0%	12	18.8%	4	14.8%	1	3.9%	3	27.3%
IgG or IgM															
Negative	0	13	44.8%	44	38.3%	33	53.2%	38	59.4%	17	63.0%	22	84.6%	6	54.6%
Positive	330	16	55.2%	71	61.7%	29	46.8%	26	40.6%	10	37.0%	4	15.4%	5	45.5%
*Subset with negative test ≤ 60 days prior*															
IgG or IgM															
Negative	0	38	52.1%	90	65.7%	58	69.1%	48	72.7%	47	79.7%	36	85.7%	31	83.8%
Positive	371	35	48.0%	47	34.3%	26	31.0%	18	27.3%	12	20.3%	6	14.3%	6	16.2%

*Defined as the first positive test

Some test results were likely false positives, making it difficult to know what portion of the early decline in test positivity was due to true sero-reversion versus simple correction of an original false positive result. To minimize the effect of false positives, we examined data from the smaller number of participants whose first positive test was positive for both IgG and IgM (specificity = 100% for both the Syntron and Teco LFAs based on National Cancer Institute (NCI) validation panels). Similar to the overall results, relatively few of these participants who were tested again in the first 30 days remained positive for both IgG and IgM (35%). Even when counting either IgG or IgM in the subsequent tests, the sero-positive rate was only 55% in the first 30 days following the initial positive test. In the second month following the initial positive test the test positive rate rose slightly to 62% but then steadily declined over the ensuing four months (Table 2).

For participants whose first test after enrollment was positive it is impossible to know how much time had passed since their primary infection. Therefore, we restricted the analysis to the 371 people whose first positive test was preceded by a negative test ≤ 60 days prior (mean, 95% confidence interval (CI) = 38.8, 37.6–40.0 days). As in the full cohort, the test positive rate declined to less than 50% within 30 days and exhibited a steady decline to < 15% over the ensuing five months (Table 2).

**Fig. 2:**
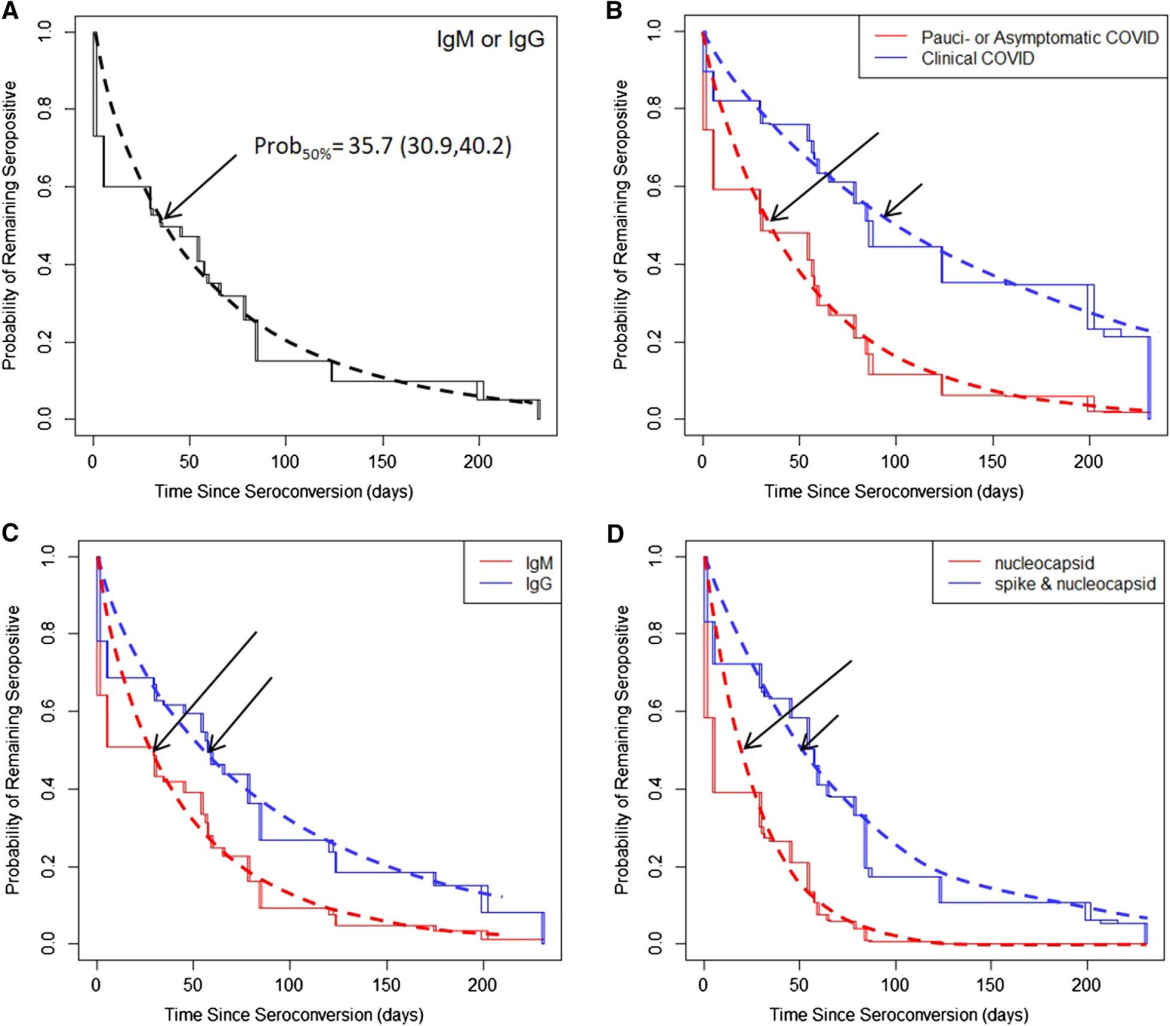
Semi-parametric and parametric (Weibull) cox proportional hazard models of sero-reversion. Prob50% indicates the parametric estimate of time when 50% of the sero-positive cohort has become sero-negative. **A** Overall rate of sero-reversion for IgM or IgG. **B** Comparison of pauci- and asymptomatic vs. clinically-defined COVID cases. **C** Comparison of rates of IgM vs IgG sero-reversion. **D** Comparison of sero-reversion rates based on follow-up testing using the Syntron test targeting antibodies to nucleocapsid proteins and the Teco test targeting a combination spike and nucleocapsid proteins

Based on analysis of the interval censored data, the estimated time to 50% sero-reversion for IgM or IgG was 35.7 days (95%CI: 30.9, 40.2) (Fig. 2A). The rate of sero-reversion was not associated with age, sex, race/ethnicity, healthcare worker status or site of enrollment. The estimated time to sero-reversion was significantly faster in participants who were pauci- or asymptomatic compared with those with clinically diagnosed COVID (34.2 days (95%CI:29.6, 39.0) vs 99.3 days (95%CI:34.8, 154.5); Cox model Hazzard Ratio (HR) ± standard error (SE) = 0.36 ± 0.24, p = 2.8 × 10^–5^, Fig. 2B). As expected, the duration of the IgM response was significantly shorter than the IgG response (27.0 days (95%CI: 23.0, 31.5) vs 54.9 days (95%CI:43.8, 64.9), Cox model HR ± SE = 0.55 ± 0.09, p = 2.3 × 10^–10^, Fig. 2C).

Likewise, based on the antigen targets used by the two assays documenting time to sero-reversion, the humoral response to the nucleocapsid antigens (Syntron) was significantly shorter than the response to a combination of spike and nucleocapsid antigens (Teco) (18.6 days (95%CI: 21.5, 30.7) vs 49.8 days (95%CI: 72.4, 150.7), Cox model HR ± SE = 0.32 ± 0.11, p = 4.4 × 10^–16^, Fig. 2D). In subset of participants whose initial test was positive for both IgG and IgM the estimated time to 50% sero-reversion was more prolonged (78.0 days; 95%CI: 33.2,123.6), albeit with somewhat wider confidence limits due to a smaller sample size (data not shown).

## Discussion

In this study detectable antibody responses to SARS-CoV-2 in a largely pauci- or asymptomatic cohort were short-lived. Most cases sero-reverted in ~ 30 days following documented sero-conversion. These data suggest that cross-sectional COVID-19 sero-surveillance studies may have underestimated the population prevalence of prior infection [2, 5, 19–26]. This observation has important implications for the epidemiology SARS-CoV-2. It suggests that community transmission of this pathogen may be even greater than currently presumed. By extension, estimates of hospitalization rate, infection fatality ratio and other measures of virulence, may also need to be revised downward. This in no way diminishes the magnitude of effect of this virus on public health. It simply highlights how pathogens causing morbidity and mortality in only a small percentage of cases can still pose a serious threat to public health when wide-spread community transmission occurs.

Not only does the short duration of elevated antibodies in minimally symptomatic cases make them difficult to discover, it also raises a question about their long-term immunity. The answer to this question could have important implications for general public health interventions as well as the timing and targeting of population-wide interventions [27]—especially since the number of cases with an abbreviated humoral response is likely to be quite high. More data are needed on memory B- and T-cell generation and protection from re-infection in this large group of people with a clinically silent infection accompanied by a relatively brief humoral response [28, 29].

Recently, Lumley et.al reported results of longitudinal sero-surveillance in 452 healthcare workers following an initial positive SARS-CoV-2 serology result [30]. Similar to the current study, they documented relatively rapid decay in IgG antibody titers over a period of several months, although a direct comparison of their estimated IgG half-life using a quantitative luminescent assay (85 days) and our estimate of IgG sero-positivity based on qualitative lateral flow assays (55 days) is not possible without a calibration of the lateral flow assays against the quantitative immunoassay. Importantly, in the UK study 61% of their participants recalled prior COVID-like symptoms and 21% had a positive SARS-CoV-2 PCR test as a result of symptomatic testing compared with the current study cohort which included predominately asymptomatic cases based on active daily symptom surveillance. In a separate study from the United Kingdom (UK) Ward et.al. reported declining rates of sero-positivity based on three distinct cross-sectional population-based surveys from June to September 2020 [31] Although the sample size in this UK study was considerably larger than the current study, the absence of longitudinal data in the same subjects make it difficult to separate the effects of declining rates of detectable antibodies from changes in the background rate of new infections.

Numerous studies indicate that the severity of the clinical illness may influence the duration of the humoral response. Most of the data comes from small laboratory studies of people with clinically evident infections [28, 32]. Information on the kinetics of antibody responses in pauci- and completely asymptomatic cases are based on small samples sizes and considerable variability in the definition of pauci- or asymptomatic case status and the duration of follow-up [8,9,37,38,39,40]. Nevertheless, these laboratory studies generally report that people with milder disease have a lower peak and a more rapid decline of SARS-CoV-2 specific IgG or IgM antibodies than more symptomatic cases. In the current study, less than 5% of the study participants had a COVID-like illness (fever plus shortness of breath or cough) and more than 70% (n = 843) reported no symptoms in the 30 days prior to their first positive test. Importantly, these asymptomatic cases represent a large faction of all cases in the population. Understanding the humoral dynamics in these people is essential when using serologic testing to characterize the dynamics of the pandemic.

Typically IgM antibodies are more transient than IgG, similar to what was observed in our data. However, some data also suggest that the humoral response may also be influenced by the antigen target. Ripperger et al. [4] found that levels of IgG to the spike proteins (S2 and receptor binding domain) remained elevated much longer and more consistently than to the nucleocapsid proteins, including among volunteers with few or no symptoms. Our study also provides evidence of a more durable IgG and IgM response targeting spike and nucleocapsid versus exclusively nucleocapsid proteins.

The sample size in the current study allowed us to test for differences in time to sero-reversion as a function of age, sex, and race/ethnicity. Interestingly, among our mostly pauci- and asymptomatic cases none of these factors were related to time to sero-reversion. This is in contrast to associations between age and race/ethnicity and risk for symptomatic infection [41, 42]. Understanding the factors that are associated with pauci- or asymptomatic infection and an abbreviated humoral response and clinically symptomatic disease with a more durable humoral response may provide novel insights about virology, immunology and clinical medicine with implications that extend beyond the current pandemic.

There are several limitations of our study. First, the sampling frame (two large healthcare system patient populations) and participants (volunteers) may reflect various biases including response bias that could be related to rates of sero-conversion and sero-reversion in unknown directions. The preponderance of white participants and more female than male participants in the current study also raises questions about the generalizability of the results, although within the limits of statistical power afforded by the sample size, there were no clear difference in rates of sero-reversion by age, race/ethnicity or sex.

The serology tests employed in this study were qualitative lateral flow assays with less than perfect sensitivity and specificity based on the FDA Emergency Use Authorization evaluation process, which itself is limited because of the modest number of cases and pre-pandemic controls used for validation. As a result, there were undoubtedly some false positives and false negatives which may have influenced the apparent rate of sero-reversion. Depending on the (unknown) number of false positives and false negatives, the rate of sero-reversion could be biased to appear shorter than it really is. To address this concern we, performed an additional analysis among a much smaller number of individuals whose first positive test was less likely to be a false positive (positive for both IgG and IgM). Here the time to sero-reversion was indeed longer—the estimated time for 50% of individuals to become completely negative was 78 days, which is still consistent with a pattern of rapidly declining seropositivity in a population-based sample of largely asymptomatic cases. The overall pattern of declining sero-positivity and the differences between clinically evident and clinically silent infections has important implications concerning the large amount of cross-sectional serologic data that have been generated to evaluate the dynamics of the pandemic.

Small differences in the test performance of the two assays (Syntron vs Teco) could also have affected the comparison of sero-reversion rates between antibodies to nucleocapsid vs spike or nucleocapsid antigens. However, the differences in sensitivity and specificity between the two assays are small and the results are consistent with prior (smaller) studies comparing responses of nucleocapsid versus spike directed antibodies.

Finally, the study design and contemporary factors related to the pandemic did not permit a regularly scheduled cadence of testing. Nevertheless, the data include a large number of tests with a continuous distribution over a wide period of time following an initial positive test allowing for good resolution in the estimates of time to sero-reversion. The COVID-19 Community Research Partnership has expanded to eight other medical centers to recruit additional participants for ongoing longitudinal surveillance. This will provide more data on antibody dynamics in primary infections and following vaccinations and support long-term clinical follow-up of asymptomatic cases to answer fundamentally important questions about how duration of initial antibody responses relate to the degree of subsequent protection from re-infection.

## Conclusions

These data document the duration of detectable antibody responses in a large number of mostly asymptomatic and minimally symptomatic cases of COVID-19. The short duration of the humoral response suggests that the true population prevalence of prior SARS-CoV-2 infection is likely significantly higher than presumed based on earlier sero-surveillance studies. The impact of the large number of cases with minimal symptoms and abbreviated antibody responses on population immunity remains to be determined.

### Supplementary Information

**Additional file 1: Figure S1.** Distribution of longitudinal testing among participants that sero-converted vs. those that remained negative during the period of follow-up.

## Data Availability

The datasets used and/or analysed during the current study are available from the corresponding author on reasonable request.

## Appendix

The COVID-19 Community Research Partnership Study—Writing Group

David M Herrington^1^

Thomas F Wierzba^2^

Martha Alexander-Miller^3^

Mark Espeland^4^

Alain G. Bertoni^5^

Allison Mathews^6^

Austin L. Seals^1^

Iqra Munawar^2^

Michael S. Runyon^7^

Lewis H. McCurdy^8^

Michael A. Gibbs^7^

Karen Kotloff^9^

DeAnna Friedman-Klabanoff^9^

William Weintraub^10^

Adolfo Correa^11^

Diane Uschner^12^

Sharon Edelstein^12^

Michele Santacatterina^12^

^1^Section on Cardiovascular Medicine, Department of Medicine, Wake Forest University School of Medicine, Winston Salem, NC, USA

^2^Section on Infectious Diseases, Department of Medicine, Wake Forest University School of Medicine, Winston Salem, NC, USA

^3^Department of Microbiology and Immunology, Wake Forest University School of Medicine, Winston Salem, NC, USA

^4^Section on Geriatrics, Department of Medicine, Wake Forest University School of Medicine, Winston Salem, NC, USA

^5^Department of Epidemiology, Division of Public Health Sciences, Wake Forest University School of Medicine, Winston Salem, NC, USA

^6^Department of Sociology, Division of Public Health Sciences, Wake Forest University School of Medicine, Winston Salem, NC, USA

^7^Department of Emergency Medicine, Atrium Health, Charlotte, NC, USA

^8^Department of Infectious Diseases, Atrium Health, Charlotte, NC, USA

^9^Center for Vaccine Development, University of Maryland School of Medicine, Baltimore, MD 21201-1509, USA.

^10^Section of Interventional Cardiology, MedStar Washington Hospital Center, Washington, DC, USA.

^11^Departments of Medicine and Pediatrics, University of Mississippi Medical Center, Jackson, MS, USA.

^12^Biostatistics Center, Milken School of Public Health, George Washington University Biostatistics Center, Rockville, MD, United States.

## Abbreviations

WFBH: Wake Forest Baptist Health
AH: Atrium health
PCR: Polymerase chain reaction
NCI: National Cancer Institute
CI: Confidence interval
HR: Hazard ratio
SE: Standard error
UK: United Kingdom
