# Supplementary material for: Duration of SARS-CoV-2 sero-positivity in a large longitudinal sero-surveillance cohort: the COVID-19 Community Research Partnership
Source: BMC Infect Dis. 2021 Aug 30;21:889. doi: 10.1186/s12879-021-06517-6 (PMC8404407; doi:10.1186/s12879-021-06517-6)
Supplement: Supplementary file 1 — Additional file 1: Figure S1. Distribution of longitudinal testing among participants that sero-converted vs. those that remained negative during the period of follow-up. [file 12879_2021_6517_MOESM1_ESM.docx]

**Duration of SARS-CoV-2 Sero-Positivity in a Large Longitudinal Sero-Surveillance Cohort: The COVID-19 Community Research Partnership**

**Authors:** The COVID-19 Community Research Partnership Study Group

**Running Title**: Duration of SARS-CoV-2 Sero-Positivity

**Corresponding Author:**

David Herrington, MD, MHS

Wake Forest University School of Medicine

Medical Center Blvd.

Winston Salem, NC 27157

[dherring@wakehealth.edu](mailto:dherring@wakehealth.edu)

336-716-4950 (office)


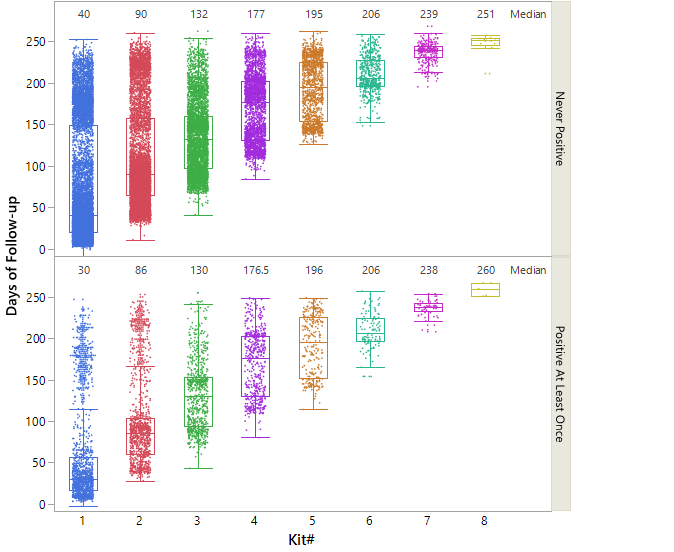
**Additional Material**

**Figure S1**. Distribution of longitudinal testing among participants that sero-converted vs. those that remained negative during the period of follow-up.
